# Metachronous or synchronous male breast and prostate cancers a duality to lookout for.

**DOI:** 10.12688/f1000research.16997.2

**Published:** 2019-02-26

**Authors:** Alain Mwamba Mukendi, Eunice Van Den Berg, Sugeshnee Pather, Rushen Siva Padayachee

**Affiliations:** 1 Department of Urology, Chris Hani Baragwanath Academic Hospital / University of the Witwatersrand, Johannesburg, South Africa; 2 Division of Anatomical Pathology, National Health Laboratory Service/Chris Hani Baragwanath Academic Hospital, Faculty of Health Sciences, University of the Witwatersrand., Johannesburg, South Africa

**Keywords:** Male breast cancer; prostate cancer; duality; metachronous; synchronous; men of African descent.

## Abstract

**Introduction**: Breast cancer is well known as the stereotypical women's cancer, and prostate cancer represents the well-known stereotypical male counterpart. While prostate cancer carries the potential to metastasize to the breast, the synchronous or metachronous co-occurrence of primary breast and primary prostate cancers is quite unusual. Prostate cancer in men of African descent may have its own behaviour with regards to its relationship with male breast cancer.

Case presentation:

Case 1: A 64 year old male presented to Chris Hani Baragwanath Hospital (CHBAH) with a 2 years history of a painless left breast lump. A core biopsy was done and confirmed breast carcinoma. Tamoxifen was started but, due to disease progression, he underwent left modified radical mastectomy followed by chemotherapy. Prostate biopsy was done for raised Prostate Specific Antigen (PSA) and suspicious prostate on digital rectal examination. A prostatic adenocarcinoma was subsequently diagnosed with bone metastases on bone scan. He was started on Androgen deprivation therapy and followed up every 3 months.

Case 2: A 68 year old male presented to CHBAH with a 1 year history of a painless right breast lump. A core biopsy confirmed breast cancer. Tamoxifen was started, followed by right modified radical mastectomy and chemotherapy for disease progression. A raised PSA and suspicious prostate on digital rectal examination prompted a prostate biopsy revealing a prostatic adenocarcinoma. Bone scan was negative for metastasis. He is currently on 3 monthly Androgen deprivation therapy and awaiting radiation.

**Conclusion**: This clinical practice article not only presents this exceptionally rare duality but highlights that both cancers can coexist either as sporadic conditions, or as a result of genetic mutations. Thus, we suggest that men with prostate cancer be screened clinically, biochemically and genetically for breast cancer and vice versa.

## Introduction

Prostate cancer is one of the leading causes of male cancer death ^1^. It is the most common conditions seen in patients in our outpatient department. It represents about one third of conditions encountered on a daily basis, out of about 200 consultations.

Male breast cancer is very rare, and comprises only about 1% of all breast cancers ^1, 2^. Prostate cancer and breast cancer synchronous or metachronous duality is quite unusual ^3^. Most of the reported cases of this dual pathology were prostate cancer patients on estrogen therapy who later on developed breast cancer ^4, 5^.

Men with a family history of breast or prostate cancer have elevated prostate cancer risks, including risk of very aggressive disease ^6^. Sporadic cases are exceptionally rare ^1^.

We present 2 cases of primary prostate and primary breast cancers in patients of African descent, who were diagnosed first with breast cancer without any family history of breast cancer, and then with prostate cancer. This is the first such case series from Africa.

## Case presentation

### Case 1

A 64 year old black male, retired forensic pathology auditor, was referred to urology clinic at CHBAH from medical oncology at the end of June 2018 with a prostate specific antigen (PSA) of 43.82 ng/dL. His hospital attendance had begun in October 2016 when he presented to the breast unit with a 2 years history of a painless progressively enlarging left breast lump with further investigations revealing carcinoma of the left breast. He was diabetic, hypertensive on treatment, and HIV negative. There was no known family history of breast, prostate or any other cancers. There was no history of undescended testes, testicular injury or mumps orchitis. A left modified radical mastectomy was performed in April 2017. He was previously a smoker, smoking 6 cigarettes per day for 40 years (12 pack-year), and quit in May 2017.

On physical examination, performed in June 2018, scars from left mastectomy and axillary lymph nodes dissection were noted. He had a normal right breast with no palpable lumps. Digital rectal examination revealed an approximately 30 g firm prostate with a nodule on the left lobe. The rest of the examination was unremarkable.

### Investigations and results

***Mammography (October 2016).*** Mammograms showed the left breast was Breast Imaging Reporting and Data System (BIRADS) category 6 and BIRADS category 1 for the right breast.

***Core biopsy of the left breast (October 2016).*** Pathological diagnosis: An invasive carcinoma of no special type.

Immunohistochemistry: Estrogen receptors: Strong positivity in 67–100% of cells.

Progesterone receptors: Strong positivity in 34–66% of cells. HER2neu: Score 1+ Negative. Ki-67: 20%

***Left breast and axillary dissection histopathology assessment (April 2017).*** Pathological diagnosis: Infiltrating duct carcinoma. On macroscopic examination the left breast nipple and skin were unremarkable. On section a tumour was noted in the lower outer and inner quadrants. It measured 40mm in maximal diameter. Microscopically, the tumour comprised infiltrating epithelioid cells in a nested growth pattern, with some tubule formation. There was marked nuclear pleomorphism, and eight mitotic figures were noted in ten high power fields. The modified Bloom and Richardson classification was Grade 2. Five lymph nodes were identified, and all of these contained tumour metastases.

**Figure 1.**
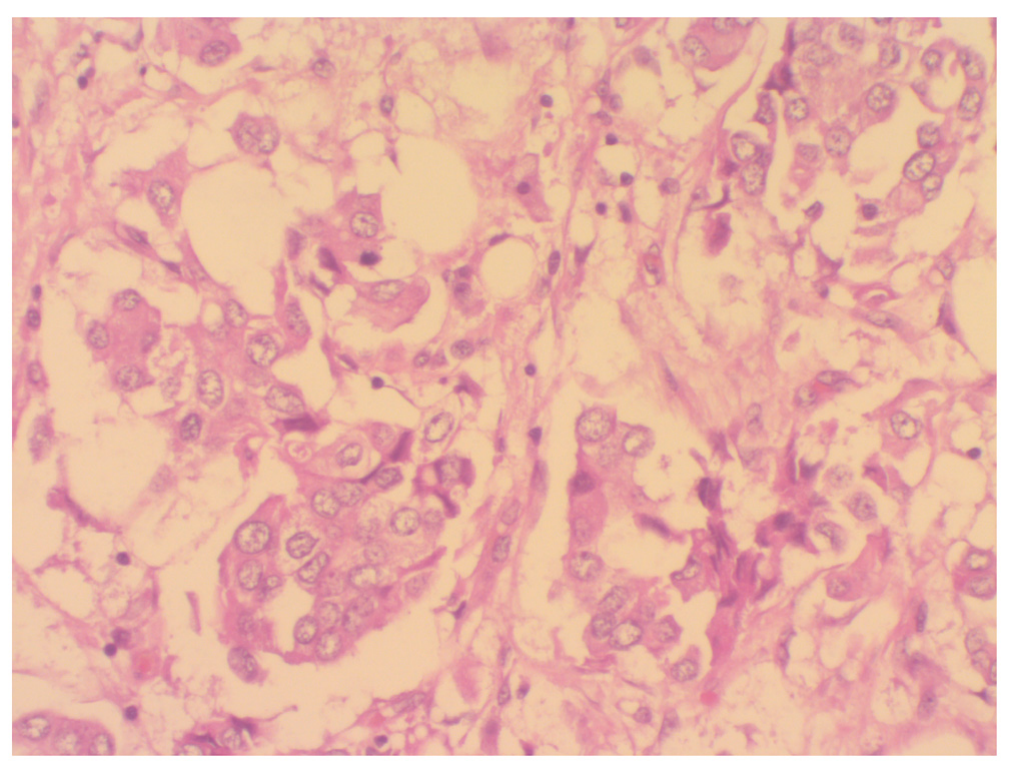
Breast carcinoma showing an adenocarcinoma with marked nuclear pleomorphism. (H&E stain, X400 magnification).

Immunohistochemical stains were positive for estrogen receptors, negative for progesterone receptors, and equivocal for HER2. FISH testing was negative for HER2 amplification. Ki67 staining showed a proliferation index of 20% ( Figure 1). The PSA immunochemistry staining was negative confirming primary breast carcinoma.

***Bloods tests.***

-Prostate specific antigen: 43.82ng/dL (normal range=0–4.5)

-Lactate dehydrogenase: 254 U/L (normal range=48–115)

-Calcium: 2.63 mmol/L (normal range=2.15–2.50)

-Alkaline phosphatase (ALP): 49 U/L (normal range=53–128)

**Figure 2.**
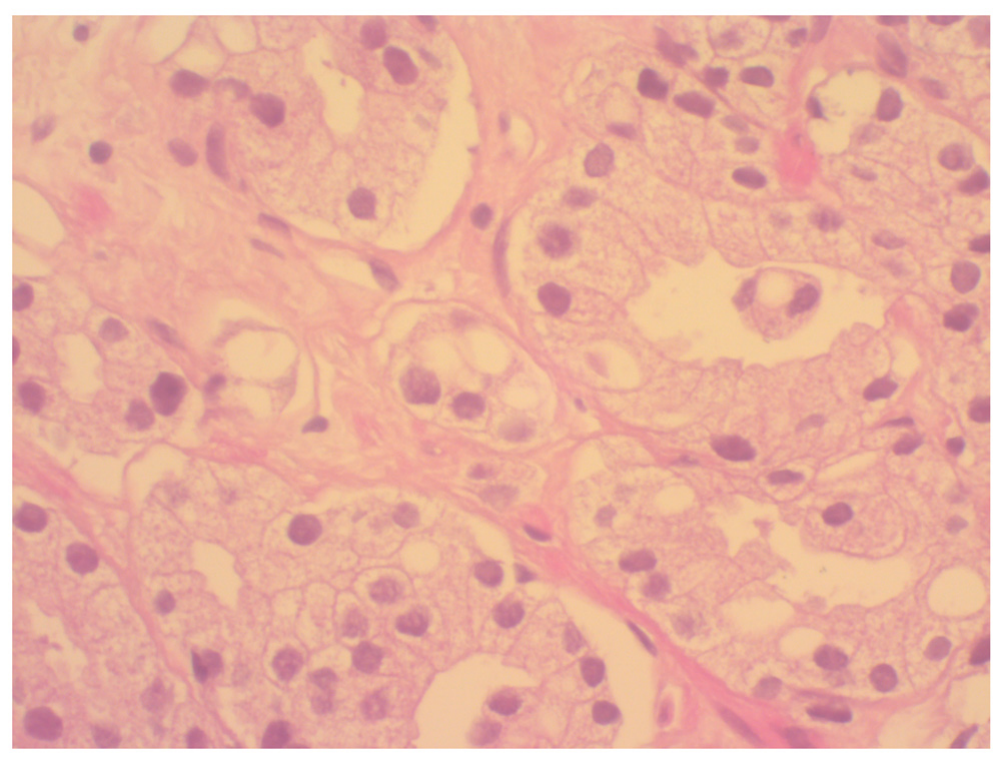
Prostatic adenocarcinoma. (H&E stain, X400 magnification).

***Prostate biopsy.*** Microscopic examination of the prostate core biopsies demonstrated an invasive prostatic adenocarcinoma. The tumour comprised predominately infiltrative, poorly formed, fused glands, and a minor component of more well differentiated glandular areas that splayed muscle fibres. The poorly formed fused glands were compatible with a Gleason pattern of 4 and the well-formed glands were compatible with a Gleason pattern of 3 (Gleason score = 7; WHO grade group 3). The tumour cells had large nuclei, conspicuous nucleoli and abundant pale eosinophilic cytoplasm ( Figure 2). The immunohistochemical profile of the tumour (CK 7 and CK20 negative; PSA monoclonal positive) confirmed primary prostatic origin.

***Computerised tomography (CT) scan of the brain, chest and abdomen.*** Global cerebral involutional changes, postsurgical changes in the left chest wall and axilla, and sclerotic lesions at L3 and right iliac bone were found on the CT scans.

***Bone scan.*** Active pathology at L3 and right iliac bone were found on Bone scan.

### Management

Initially assessed as T2N0M0 in October 2016, he was put on tamoxifen 20 mg per os daily. 6 months later (April 2017) in view of disease progression to T4bN1Mx, a left mastectomy and left axillary lymph nodes dissection was done. Tamoxifen was replaced by anastrozole 1 mg daily and He was then referred for chemo-radiation. He received 4 cycles of chemotherapy AC-T (Adriamycin, cyclophosphamide and taxol) completed in January 2018. A second primary cancer, a prostatic adenocarcinoma was diagnosed in July 2018, almost 2 years after the initial one, for which he is currently on Goserelin 10.8 mg subcutaneously every 3 months. He has so far received 2 injections (July and October of 2018) and will follow up in 3 months for the next injection, and PSA/testosterone monitoring will be done at 6 months as per the hospital policies (funds restrictions). He is unlikely to be given radiation therapy for curative intent as the disease was found to be metastatic.

### Case 2

A 68 year old black male, retired teacher, was referred to urology clinic at CHBAH in May 2018 from medical oncology with a PSA of 113 ng/dL. He first presented in December 2016 to CHBAH breast unit with a 1 year history of a painless right breast lump with further investigations revealing carcinoma of the right breast. He reported that his father died of cancer, but does not know which cancer it was. He had no medical history, was HIV negative, and had no history of undescended testes, mumps orchitis or testicular injury. He was a heavy smoker who smoked 20 cigarettes per day for 40 years (40 pack-year) and quit in July 2016. Right mastectomy and axillary lymph nodes dissection was performed in April 2017.

On physical examination, scars from right mastectomy and axillary lymph nodes dissection were observed. He had a normal left breast with no palpable lumps. Digital rectal examination revealed an approximately 40 g hard nodular prostate. The rest of the examination was unremarkable.

### Investigations and results

***Mammography (December 2016).*** Mammograms showed the left breast was BIRADS category 1 and BIRADS category 5 for the right breast.

***Core biopsy of the right breast (December 2016).*** Pathological diagnosis: Infiltrating duct carcinoma displaying cribriform features.

Immunohistochemistry: Estrogen receptor positive; strong 3+ intensity staining in >91% of tumour cells. Progesterone Receptor positive; strong 3+ intensity staining in 70% of tumour cells. HER2 negative score 1+ in approximately 10% of tumour cells. Ki-67 the tumour proliferation index approaches 30%.

**Figure 3.**
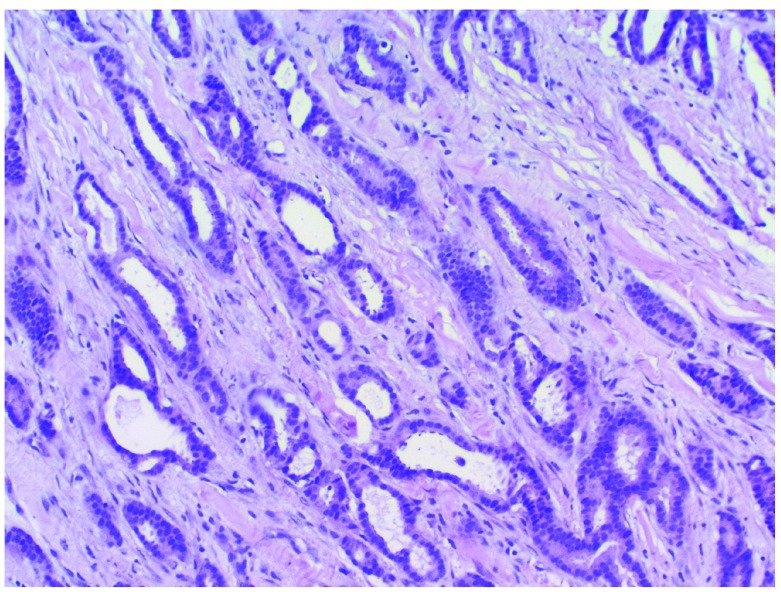
Breast infiltrating duct carcinoma (H&E stain, X200 magnification).

**Figure 4.**
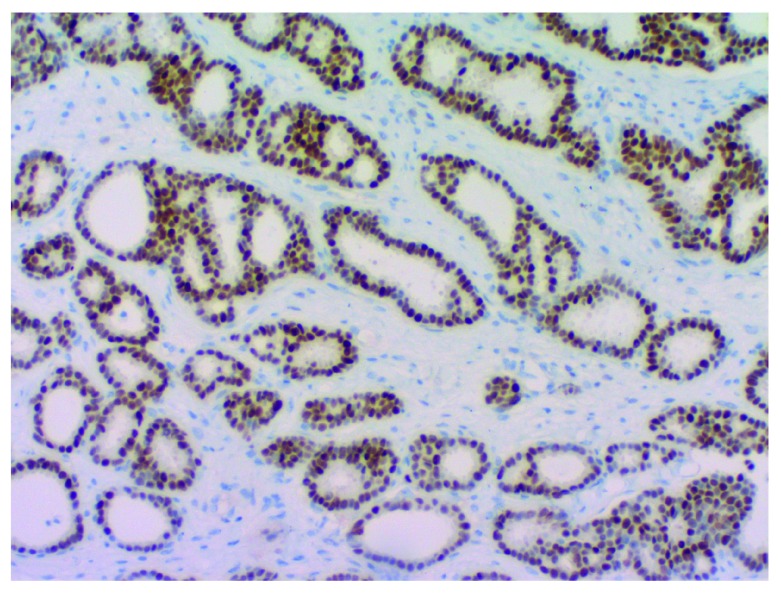
Oestrogen receptors diffusely expressed in the breast carcinoma (estrogen receptor (ER) immunohistochemistry, X200 magnification).

**Figure 5.**
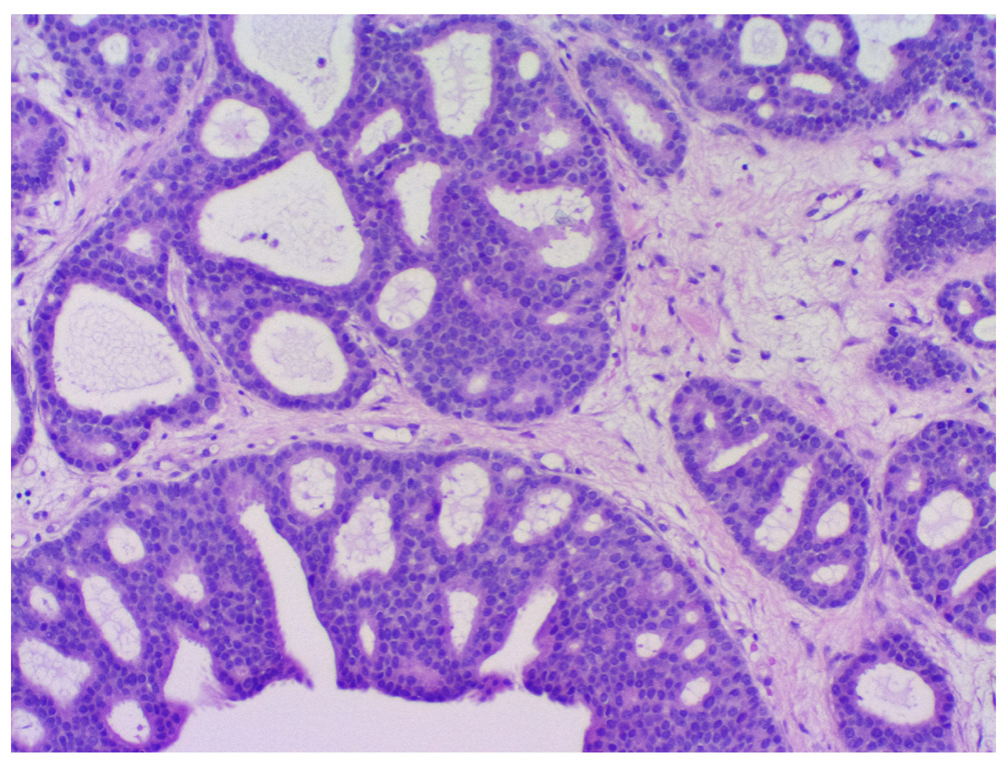
Breast cribriform ductal carcinoma *in situ* (DCIS) (H&E stain, X200 magnification).

***The right breast histopathological assessment.*** Histopathological examination showed an infiltrating duct carcinoma ( Figure 3), with a Nottingham combined histopathological grade of 1/3 (tubules 1, pleomorphism 2 and mitoses 1). Estrogen and progesterone receptors were expressed diffusely within the tumour cells ( Figure 4). Foci of low-grade cribriform ductal carcinoma *in situ* were also evident ( Figure 5). The PSA immunochemistry staining was negative confirming primary breast carcinoma.

***Bloods tests.***

-Prostate specific antigen: 113ng/dL (normal range=0–4.5)

-Lactate dehydrogenase: 289 U/L (normal range=48–115)

-Calcium: 2.56 mmol/L (normal range=2.15–2.50)

-ALP: 73 U/L (normal range=53–128)

**Figure 6.**
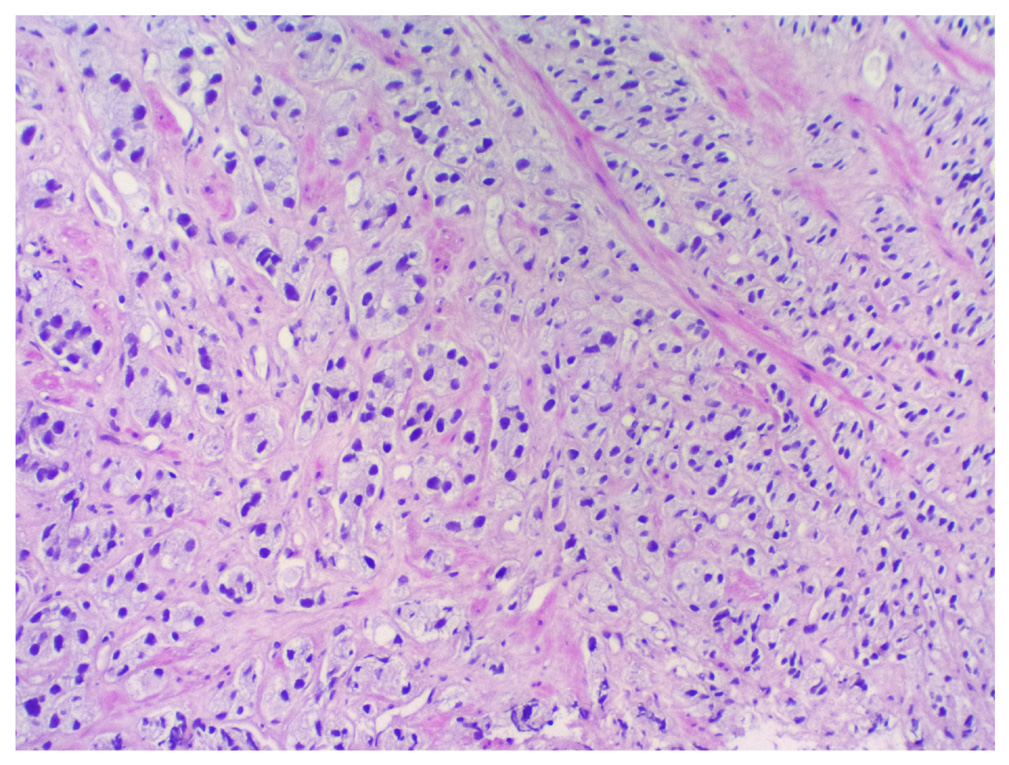
Invasive prostatic adenocarcinoma (H&E stain, X200 magnification).

**Figure 7.**
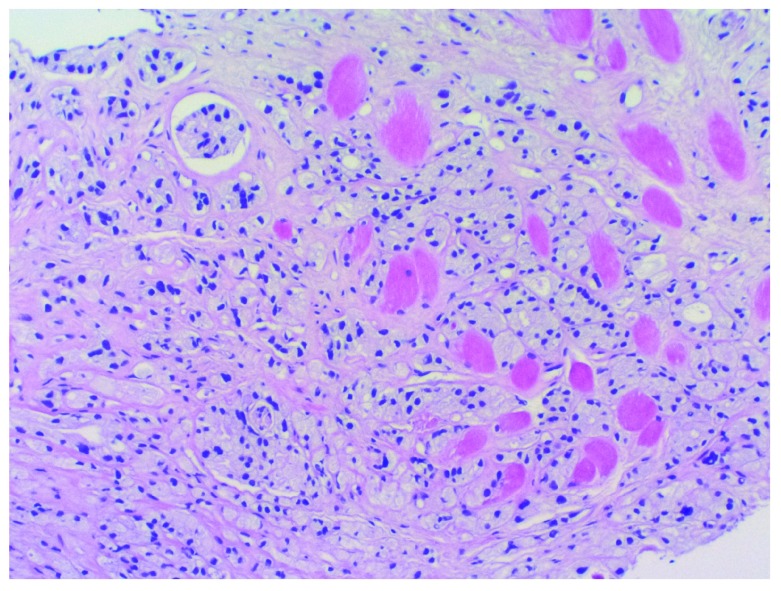
Invasive prostatic adenocarcinoma infiltrating into skeletal muscle (H&E stain, X200 magnification).

***Prostate biopsy.*** Biopsy results showed an invasive prostatic adenocarcinoma ( Figure 6), with a modified Gleason score of 9, and grade group 5, which was infiltrating into skeletal muscle ( Figure 7). The proportion of tumour infiltration was >90% within the cores. There were areas of lymphatic invasion and perineural invasion. 

***Bone scan.*** No evidence of osteoblastic metastases was found.

### Management

He was assessed from the beginning in December 2016 as T4bN0Mx, he was put on tamoxifen 20 mg per os daily. 4 months later (April 2017), due to disease progression on tamoxifen from T4bN0Mx to T4bN1Mx, a right mastectomy and right axillary lymph nodes dissection was done prior to referring him for chemo-radiation. He received 6 cycles of chemotherapy FAC, (Fluorouracil, Adriamycin and cyclophosphamide) completed in January 2018; he is still on tamoxifen. A second primary non metastatic cancer arising from the prostate, a prostatic adenocarcinoma, was diagnosed in May 2018, 17 months after the first primary. He is currently on Goserelin 10.8 mg subcutaneously every 3 months and has already received 2 doses. He is awaiting radiation therapy to the right chest. PSA/testosterone monitoring will be performed at 6 months as per the hospital policies (funds restrictions). Radiation oncologist will consider external beam radiotherapy at the time of radiation for right chest.

## Discussion

Prostate cancer is the most common male cancer in the U.S. and second leading cause of cancer death. Male breast cancer is very rare, and comprises only about 1% of all breast cancers ^1, 2^. Prostate cancer and breast cancer, as synchronous or metachronous dual primary cancer, is quite infrequent ^3^. They are both part of the hereditary breast and ovarian cancer (HBOC) syndrome which is associated with gene mutations in *BRCA1* and *BRCA2* ^7^. The *BRCA* alteration can be inherited from either of the parents and be responsible for a familial type of breast and prostate cancers which has also been previously reported ^6, 8^.

*BRCA2* and *BRCA1* mutation carriers have respectively 40% and 20% lifetime risk of prostate cancer, whereas the lifetime risk of male breast cancer is approximately 5% to 10%, and 1% to 5% for *BRCA2* and *BRCA1* mutation carriers, respectively ^9^. Mutations in *BRCA2*, in particular, have been associated with more aggressive clinicopathologic characteristics of prostate cancer and worse outcomes ^7^. The *BRCA* germline mutations in male cancers are found in only 1% – 2 % of sporadic prostate cancer ^10^. However, prostate and male breast cancers have some similarities with regards to etiology and therapeutic approaches ^2, 9, 11^.

Prostate cancer carries the potential to metastasize to the breast ^3^. Male primary breast cancer and breast metastases from prostatic carcinoma may have similar histological features ^12^. Therefore it is crucial to exclude breast metastases from prostatic adenocarcinoma through a thorough histopathological assessment. PSA immunohistochemistry may help to distinguish between primary and metastatic disease within the breast specimen. This step is very important because of its therapeutic and prognostic implications ^12^. Breast specimens from our 2 cases were evaluated for possible prostatic metastases using PSA immunochemistry staining and were both negative.

Androgen deficiency or excess estrogen increases the risk of male breast cancer in men with a history of testicular injury, mumps orchitis, or undescended testes ^3^. Klinefelter's syndrome also increases the risk of male breast cancer. Estrogen treatment for prostate cancer was reported as another risk factor for the development of male breast cancer ^3, 4^. The latter risk factor is associated with most of the reported cases of this dual pathology ^4, 5^. None of these risk factors were found in our 2 cases.

Metachronous or synchronous occurrences of both breast and prostate cancer are scarce in the current literature. Lee *et al.* evaluated 161 male breast cancer patients for prostate cancer between 1977 and 2000, and found that only 10 patients were also diagnosed with prostate cancer. Breast cancer was the first primary tumour in 8 of these 10 patients ^3^. The association of a sporadic male breast cancer with any other primary tumours, including prostate cancer, is exceptionally uncommon ^1, 9^. As with our 2 patients, just a few cases of this combination without any family history of breast cancer or any genetic mutations have been reported ^13– 16^. Treatment for each cancer includes hormone manipulation, which may increase the risk for subsequent cancers ^17^. There is no available guideline for treatment of this association, hence further research is necessary.

This is in our knowledge the first reported cases from patients of African descent living in Africa. Unfortunately, we could not evaluate our patients in terms of genetics because of the lack of some technical facilities and funds restrictions. Prostate cancer from men of African descent may have its own behaviour with regards to its relationship with male breast cancer as seen in our 2 cases, both are high risk for metastases but one seems to be more aggressive than the other one.

Prostate cancer is in general a slow growing cancer and on that account many years have to pass before developing any symptomatology ^8^. We strongly believe that in those 2 patients even though prostate cancer was the second diagnosed primary they both coexisted undiagnosed at some point in time. Therefore it is absolutely difficult to say which one started first. Most likely if prostate cancer screening was done when they were in their 50s, prostate cancer would have been detected first. 

## Conclusion

Breast and prostate cancer as synchronous or metachronous primary cancers are very rare and can be either sporadic or as a result of genetic mutations. When either a breast or prostate cancer is diagnosed in a male patient, clinicians should be alert for the other, however, because such a co-occurrence is rarely diagnosed or reported this rarely happens. More studies and research may be helpful to understand the behaviour of these synchronous or metachronous breast and prostate cancers particularly in an African setting.

A suggestion is therefore made that men with prostate cancer be screened clinically, biochemically and genetically for breast cancer, or vice versa, in view of possible common genetic factors contributing to the pathogenesis of both cancers.

## Take-away lessons

- Synchronous or metachronous Breast and prostate cancer are rarely reported, and sporadic cases are extremely rare.
- BRCA mutation is found to be a common genetic pathway in both cancers.
- Because of the possible link between the 2, clinicians should lookout for this duality regardless of which one is diagnosed, being investigated or worked up first.
- It is crucial to do PSA staining on both specimens (breast and prostate) to exclude metastases.

## Consent

Written informed consents were obtained from both patients for publication of this manuscript and accompanying results.

## Data availability

All data underlying the results are available as part of the article and no additional source data are required.
