# Supplementary material for: Metachronous or synchronous male breast and prostate cancers a duality to lookout for
Source: F1000Res. 2019 Feb 26;7:1825. Originally published 2018 Nov 20. [Version 2] doi: 10.12688/f1000research.16997.2 (PMC6619383; doi:10.12688/f1000research.16997.2)
Supplement: Peer review history

# Peer review: Reviewer response for version 2

**DOI:** 10.5256/f1000research.20122.r44973

**Published:** 2021-06-16

**Authors:** Pankaj Joshi, Sandeep Bafna

**Affiliations:** 1 Centre for Reconstructive Urology, Pune, Maharashtra, India; 2 Centre for Reconstructive Urology, Pune, Maharashtra, India

I do not have any issue with the final edited version. Have gone through all the changes requested earlier.

Are enough details provided of any physical examination and diagnostic tests, treatment given and outcomes?

Yes

Is the background of the cases’ history and progression described in sufficient detail?

No

Is sufficient discussion included of the importance of the findings and their relevance to future understanding of disease processes, diagnosis or treatment?

No

Is the conclusion balanced and justified on the basis of the findings?

No

Reviewer Expertise:

Reconstructive Urology

We confirm that we have read this submission and believe that we have an appropriate level of expertise to confirm that it is of an acceptable scientific standard.

# Peer review: Reviewer response for version 2

**DOI:** 10.5256/f1000research.20122.r50324

**Published:** 2019-07-08

**Authors:** Celestin Nzanzu Mudogo

**Affiliations:** 1 Institute for Biochemistry and Molecular Biology, University of Hamburg, Hamburg, Germany

Strength of the paper

- First case reported on duality between breast and prostate cancer in male. This finding may suggest a specific prostate surveillance or genetic screening for men prostatism symptoms or asymptomatic rise in PSA.

Weakness of the research work

- Lack of genetic testing outcomes correlation to the clinical cases presented still a limitation of this study.
- Some studies suggest an evidence correlation between Hormone and symptomatic or asymptomatic prostate cancer! Unfortunately, the authors could not follow up and present the Hormones profiles before and after the Tamoxifen treatment and Prostate cancer diagnosis because of funding issues!  This could be very informative to be discussed and need to be consider in their future work.

In sum, these rare cases reported is an initial presentation of the duality between prostate to breast cancer in men! I recommend the paper for indexing as a clinical case report and I´m looking forward to reading the next paper based on future work discussed in this paper.

Are enough details provided of any physical examination and diagnostic tests, treatment given and outcomes?

Partly

Is the background of the cases’ history and progression described in sufficient detail?

Yes

Is sufficient discussion included of the importance of the findings and their relevance to future understanding of disease processes, diagnosis or treatment?

Partly

Is the conclusion balanced and justified on the basis of the findings?

Yes

Reviewer Expertise:

Biochemistry and Molecular Parasitology

I confirm that I have read this submission and believe that I have an appropriate level of expertise to confirm that it is of an acceptable scientific standard, however I have significant reservations, as outlined above.

# Peer review: Reviewer response for version 2

**DOI:** 10.5256/f1000research.20122.r44974

**Published:** 2019-03-18

**Authors:** Bannakij Lojanapiwat

**Affiliations:** 1 Department of Surgery, Chiang Mai University, Chiang Mai, Thailand

I do not have changes to the final version that it is ok for indexing.

Are enough details provided of any physical examination and diagnostic tests, treatment given and outcomes?

Yes

Is the background of the cases’ history and progression described in sufficient detail?

Yes

Is sufficient discussion included of the importance of the findings and their relevance to future understanding of disease processes, diagnosis or treatment?

Partly

Is the conclusion balanced and justified on the basis of the findings?

No

Reviewer Expertise:

NA

I confirm that I have read this submission and believe that I have an appropriate level of expertise to confirm that it is of an acceptable scientific standard.

# Peer review: Reviewer response for version 1

**DOI:** 10.5256/f1000research.18583.r41758

**Published:** 2019-01-15

**Authors:** Bannakij Lojanapiwat

**Affiliations:** 1 Department of Surgery, Chiang Mai University, Chiang Mai, Thailand

Both breast cancer and prostate cancer are hormonal dependent and have genetic alteration in recent studies, This article is not perfect due to lack of genetic testing that is difficult to explain the co-incident of both cancer.

 Conclusion is quite long, author should rewrite. Conclusion has this sentence 'suggest that men with prostate cancer be screened clinically, biochemically and genetically for breast cancer and vice versa.' Maybe write 'only suggests that men with prostate cancer can be screened for breast cancer.'

 If possible summary of the case report in Table maybe more understand the natural history and treatment of this rare condition.

Are enough details provided of any physical examination and diagnostic tests, treatment given and outcomes?

Yes

Is the background of the cases’ history and progression described in sufficient detail?

Yes

Is sufficient discussion included of the importance of the findings and their relevance to future understanding of disease processes, diagnosis or treatment?

Partly

Is the conclusion balanced and justified on the basis of the findings?

No

Reviewer Expertise:

Urooncology, endourology

I confirm that I have read this submission and believe that I have an appropriate level of expertise to confirm that it is of an acceptable scientific standard, however I have significant reservations, as outlined above.

# Peer review: Reviewer response for version 1

**DOI:** 10.5256/f1000research.18583.r40909

**Published:** 2018-12-04

**Authors:** Pankaj Joshi

**Affiliations:** 1 Centre for Reconstructive Urology, Pune, Maharashtra, India

It's an interesting observation.

 I think authors should mention BRCA Gene mutation's role in breast and prostate cancer.

 Why is the second patient on ADT if bone scan was negative?

 Details are important when discussing cancer:

 What was the breast pathology? Ductal or lobular? What were the patients' Gleason scores and PSAs?

 Which men would you screen for breast cancer since it is so rare - less than 1/100? Which genetic tests? Be clear with screening goals because you are talking about screening parameters because prostate cancer affects a massive population.

 Those are my thoughts to the author(s). It is an idea which needs refinements and more details regarding how and why we should carry their message into practice.

Are enough details provided of any physical examination and diagnostic tests, treatment given and outcomes?

Yes

Is the background of the cases’ history and progression described in sufficient detail?

No

Is sufficient discussion included of the importance of the findings and their relevance to future understanding of disease processes, diagnosis or treatment?

No

Is the conclusion balanced and justified on the basis of the findings?

No

Reviewer Expertise:

Reconstructive Urology

I confirm that I have read this submission and believe that I have an appropriate level of expertise to confirm that it is of an acceptable scientific standard, however I have significant reservations, as outlined above.

# Response

**Published:** 2018-12-04

**Authors:** Alain Mwamba Mukendi

**Affiliations:** University of the Witwatersrand / Chris Hani Baragwanath Academic Hospital, South Africa

Thank you for your review. I am not sure if you could access the entire article most of the points raised have their answers in the article. All the PSA, Gleason scores etc. Are under case presentation. The role of BRCA was also highlighted under the discussion. Both breast malignancies were ductal as shown in figures 3 and 5. We will on the next version specify that for the first case.
